# Management of a Patient with Metastatic Colorectal Cancer and Liver Metastases

**DOI:** 10.1155/2014/790192

**Published:** 2014-03-12

**Authors:** Muhammad Wasif Saif

**Affiliations:** Tufts University School of Medicine, 800 Washington Street, Suite 7S-7099, Boston, MA 02111, USA

## Abstract

Liver metastases are commonly encountered in patients presenting with metastatic colorectal cancer (mCRC); resection is the treatment of choice. A number of systemic treatment options are currently available for such patients, including the use of 5-fluorouracil-based chemotherapies and oxaliplatin (e.g., FOLFOX) in combination with biologic agents that target angiogenesis (e.g., bevacizumab). For patients with progression following first-line treatment, current second-line options include a change in chemotherapy with bevacizumab (for patients who did or did not receive prior bevacizumab) or FOLFIRI in combination with aflibercept, a more recently approved antiangiogenesis therapy. Neurotoxicity is a well-established adverse event of oxaliplatin-based therapy. The current case details an mCRC patient with liver metastases who was treated with a capecitabine and oxaliplatin regimen (XELOX), and experienced two episodes of transient cortical blindness possibly related to oxaliplatin. After disease progression, the patient was switched to a regimen of FOLFIRI and aflibercept and did well on this second-line regimen.

## 1. Introduction

In patients presenting with liver metastases in the context of metastatic colorectal cancer (mCRC), surgical resection is the treatment of choice where possible; for patients undergoing hepatic resection, five-year actuarial survival in some series ranges from 30% to over 50% [1, 2]. However, these patients remain at a high risk for relapse, with hepatic recurrence rates of up to 75% [2]. The current first-line options for systemic antitumor treatment include the use of 5-fluorouracil- (5-FU-) based therapies with oxaliplatin (e.g., FOLFOX) with or without the use of an antiangiogenesis agent (bevacizumab) [1]. The use of perioperative FOLFOX improves three-year survival rates by up to 8%, and postoperative FOLFOX can also be considered in patients with no perioperative treatment [2]. Although most patients typically present with unresectable disease, the use of regressive chemotherapies is indicated to convert patients to resectable status [1]. Indeed, the evolution of 5-FU-based chemotherapies in recent years has helped to improve resectability rates with the addition of agents such as oxaliplatin or irinotecan to these regimens [3]. Choice of pre- and/or postoperative chemotherapy depends on factors such as treatment history, patient response, and safety/toxicity issues [1]. The optimal sequence of chemotherapy relative to resection is unclear; for patients with resectable disease, perioperative treatment (neoadjuvant and postoperative) can be followed by resection, or resection can be followed by postoperative adjuvant treatment [1].

## 2. Case Presentation

The patient is a 61-year-old woman who initially presented to her primary care provider in January 2012 with some hematochezia and diarrhea accompanied by lower abdominal pain. After a change in diet failed to resolve her symptoms, she subsequently underwent a computed tomography (CT) scan, which showed severe proctocolitis. The patient was administered steroids in addition to metronidazole, but her symptoms did not resolve. She underwent a colonoscopy in June 2012, which showed the presence of a mass nearly obstructing the rectum.

On June 21, 2012, she underwent a low anterior resection of a stage IV rectosigmoid adenocarcinoma (approximately 5 × 4 × 1.5 cm). The tumor was moderately differentiated. A wedge resection of a left hepatic lobe was performed at the same time and yielded a diagnosis of adenocarcinoma consistent with the primary rectal tumor. Colostomy formation was also performed at that time. Her pathology review showed clear margins of the rectal primary, positive margin of the liver resection, and five of 15 lymph nodes positive for metastatic carcinoma. The patient tolerated the procedure well, except for bilateral extremity lymphedema starting two to three weeks after surgery, which improved with compression stockings.

A subsequent CT scan showed the presence of a secondary liver lesion as well as a 1.2 cm portacaval lymph node, positive for metastatic carcinoma with positron emission tomography (PET) CT. Otherwise, no lymphadenopathy in the chest, abdomen, or pelvis was found.

Pelvic radiotherapy was started with a total dose between 45 and 54 Gy on September 24, 2012. At about that time (September 2012), the patient was started on XELOX chemotherapy (oxaliplatin 100 mg/m^2^ over two hours, Day 1, every three weeks; capecitabine 850 mg/m^2^ orally twice daily, Days 1 to 14, every three weeks) and received approximately three cycles. Although the patient tolerated the first cycle well, the day after chemotherapy she had one isolated episode of complete blindness in her right eye, which lasted 15 seconds. A similar but shorter episode lasting between eight and 10 seconds occurred during the second cycle of XELOX. The last (third) cycle was not completed due to the occurrence of grade 2 thrombocytopenia during Cycle 2 and grade 3 thrombocytopenia during Cycle 3; capecitabine was continued without oxaliplatin during Cycle 3. Bevacizumab was also not given in combination with the chemotherapy due to the proximity of her prior liver and rectal surgery and subsequent issues with thrombocytopenia.

Restaging scans showed that after three chemotherapy cycles she appeared to have complete resolution of the one PET-avid liver lesion but persistence of the second liver lesion and the portacaval lymph node. Given those findings, as well as worsening preexisting thrombocytopenia, it was the consensus of the tumor board to give the patient additional therapy with FOLFIRI (irinotecan 180 mg/m^2^ intravenously (IV), over 90 minutes, with folinic acid 400 mg/m^2^, over 120 minutes, followed by fluorouracil 400 mg/m^2^ IV bolus, then fluorouracil 2400 mg/m^2^ IV infusion, over 46 hours) in combination with aflibercept (4 mg/kg IV infusion over one hour, every two weeks), a recently approved angiogenesis inhibitor for mCRC, which was initiated on December 17, 2012. Adverse events (AEs) occurring on the FOLFIRI/aflibercept regimen were grade 1/2 diarrhea, which was managed with loperamide and diphenoxylate/atropine, and grade 1 nausea, managed with palonosetron; a wig was also used due to alopecia. She had completed four cycles and was due for restaging scans at the end of the week but then presented for evaluation due to persistent pelvic pain following surgery. She had had stable disease; therefore, on March 26, 2013, she was sent for liver and nodal resection.

**Figure 1:**
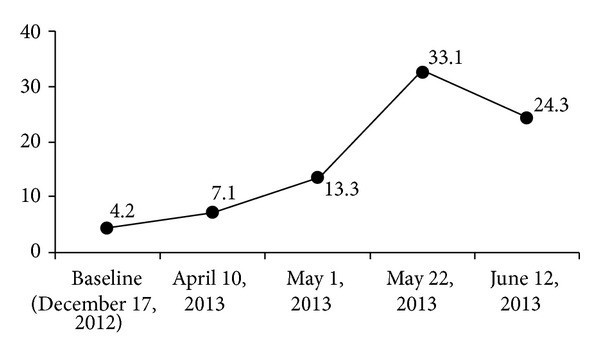
Change in carcinoembryonic antigen level (ng/mL).

The patient did quite well postoperatively and had no new complaints. After her reresection, she was started on maintenance capecitabine (1000 mg orally twice daily, Days 1 to 14, every 21 days) due to a high recurrence risk. As of May 1, 2013, she continues to do well with no evidence of disease and is being followed up for a low pelvic mass on radiography, which could reflect infection, postsurgical changes, or drop metastasis. Evolution of the patient's carcinoembryonic antigen level is shown in Figure 1.

## 3. Discussion

Metastatic liver involvement is commonly encountered in patients diagnosed with mCRC; liver metastasis is a common cause of death in these patients [1]. In patients with solitary or confined liver metastases, primary resection should be considered [2]. Improvements in perioperative morbidity and mortality over the last few decades have allowed hepatic resection to become an option for an increasing range of patients with mCRC and liver metastases [4]. The combination of chemotherapy and surgical resection is another option for the vast majority of patients who present with initially unresectable disease, in whom five-year survival is uniformly grim [5]. With the use of advanced chemotherapy, resectability rates between 12% and 22% can be achieved in these patients, with five-year survival rates of up to 35% observed after subsequent resection [3].

Oxaliplatin in combination with 5-FU or capecitabine (CapeOX or XELOX) is frequently used as first-line therapy for patients with mCRC [1, 2]. A large phase 3 study of over 2000 patients found XELOX to be noninferior to FOLFOX, with a median progression-free survival (PFS) of 8.0 and 8.5 months reported for patients in the pooled analysis for the XELOX and FOLFOX treatment arms, respectively; the corresponding median overall survival (OS) was 19.8 and 19.6 months, respectively [6, 7]. AEs were similar between XELOX and FOLFOX regimens, with similar rates of grade 3/4 neurosensory toxicity (17%), whereas hand-foot syndrome and diarrhea were more frequently observed in the XELOX arms [7]. A similar benefit of XELOX and FOLFOX in terms of curative effect has also been reported in a meta-analysis of seven randomized, controlled trials [1, 8]. Use of XELOX or FOLFOX in combination with the antiangiogenesis agent bevacizumab as first-line therapy in mCRC has been studied in the NO16966 trial; approximately 40% of patients in each treatment arm had a single metastatic site in this study. Results of NO16966 showed improvement in PFS for chemotherapy regimens (XELOX or FOLFOX) with bevacizumab compared with placebo (9.4 versus 8.0 months; hazard ratio (HR) = 0.83; *P* = 0.0023), although response rate was similar, and there was no significant benefit in OS (21.3 versus 19.9 months, resp.; *P* = 0.0769) [9]. It is believed that differences in duration of therapy and discontinuation rates may have accounted for the lack of improvement in OS in this study [1, 9].

Aflibercept, which is known as ziv-aflibercept in the United States, is a recently approved antiangiogenic agent that is designed to inhibit the vascular endothelial growth factor (VEGF) pathway by binding not only VEGF-A (like bevacizumab) but also VEGF-B and placental growth factor (PlGF) ligands. Inhibition of these endogenous ligands by aflibercept prevents binding to their cognate cellular receptors, VEGF receptor (VEGFR) 2 and VEGFR1 (in the case of VEGF-A) and VEGFR1 (in the case of VEGF-B and PlGF) [10]. This activity of aflibercept results in decreased neovascularization and decreased vascular permeability; in mice, the in vivo growth of colon tumor xenografts is inhibited by aflibercept [10]. Aflibercept is indicated in combination with FOLFIRI for patients with mCRC that is resistant to or has progressed following an oxaliplatin-containing regimen [1, 10]. Efficacy and safety of aflibercept were examined in the VELOUR trial, a randomized, double-blind, multinational study, which randomized mCRC patients (*N* = 1226) previously treated with an oxaliplatin-containing regimen to treatment with aflibercept or placebo in combination with FOLFIRI. Patients were allowed to have received prior bevacizumab, and the primary end point was OS [11]. The percentage of patients with metastatic liver involvement at baseline (exclusive of the primary site) was 70.2% and 75.0% in the placebo and aflibercept arms, respectively, and 54.9% and 57.8% of patients had >1 metastatic site [11]. Results from VELOUR demonstrated a median OS of 13.50 months for patients in the aflibercept plus FOLFIRI arm compared with 12.06 months for patients in the placebo plus FOLFIRI arm (HR = 0.817; *P* = 0.0032). Significant improvements in PFS (HR = 0.758; *P* < 0.0001) and response rate (*P* < 0.001) were also observed [11]. VEGF-related grade 3 and grade 4 AEs included hypertension, hemorrhage, arterial thromboembolic events, and venous thromboembolic events [11]. Leukopenia, diarrhea, neutropenia, proteinuria, increased aspartate aminotransferase, stomatitis, fatigue, thrombocytopenia, increased alanine aminotransferase, hypertension, decreased weight, decreased appetite, epistaxis, abdominal pain, dysphonia, increased serum creatinine, and headache were the most commonly observed AEs with aflibercept (all grades ≥20% incidence, ≥2% between arm difference). In the present case, the patient did quite well on the aflibercept plus FOLFIRI regimen, with minimal (grades 1 and 2) AEs. She achieved a possible cure from the therapy, although she will require additional follow-up.

Although not routinely administered, the use of fixed-dose maintenance capecitabine therapy in this patient is appropriate to reduce her recurrence risk while allowing for improved quality of life [12]. In a retrospective study of 28 patients receiving maintenance capecitabine for gastrointestinal malignancies, the first 11 patients received capecitabine 1000 mg twice daily without interruption; an abbreviated schedule of five days per week was used in eight patients who developed hand-foot syndrome and in the remaining patients [12]. In this study, two patients with mCRC were receiving XELOX, and two patients with gastric cancer were receiving oxaliplatin with capecitabine. Grade 1 or 2 fatigue (*n* = 16; 57%), hand-foot syndrome (*n* = 20; 71%; one grade 3), and diarrhea (*n* = 6; 21%) were the most commonly observed events; there were no grade 4 events [12]. Toxicities were not different according to age or gender. Dose reductions, most commonly for hand-foot syndrome, fatigue, and abdominal pain, were required in 11 patients (32%).

In the current case, the most likely cause of cortical blindness was the use of oxaliplatin. Neurotoxicity is the best-known event associated with the use of oxaliplatin-based chemotherapy, although occurrence of cortical blindness is rare. Reversible posterior leukoencephalopathy syndrome has been reported in clinical trials (incidence <0.1%) and in postmarketing experience with oxaliplatin and may be associated with visual disturbances ranging from blurriness to blindness [13]. The occurrence of cortical blindness has also been reported as a rare neurotoxic AE associated with the use of contrast-enhanced CT [14].
